# Prediction of sentinel lymph node metastasis in breast cancer by using deep learning radiomics based on ultrasound images

**DOI:** 10.1097/MD.0000000000035868

**Published:** 2023-11-03

**Authors:** Chujun Wang, Yu Zhao, Min Wan, Long Huang, Lingmin Liao, Liangyun Guo, Jing Zhang, Chun-Quan Zhang

**Affiliations:** a Department of Ultrasound, The Second Affiliated Hospital of Nanchang University, Nanchang, China; b Department of Information Engineering, Nanchang University, Nanchang, China; c Department of Oncology, The Second Affiliated Hospital of Nanchang University, Nanchang, China.

**Keywords:** artificial intelligence, breast cancer, deep learning, sentinel lymph node metastasis, ultrasonography

## Abstract

Sentinel lymph node metastasis (SLNM) is a crucial predictor for breast cancer treatment and survival. This study was designed to propose deep learning (DL) models based on grayscale ultrasound, color Doppler flow imaging (CDFI), and elastography images, and to evaluate how DL radiomics can be used to classify SLNM in breast cancer. Clinical and ultrasound data of 317 patients diagnosed with breast cancer at the Second Affiliated Hospital of Nanchang University were collected from January 2018 to December 2021 and randomly divided into training and internal validation cohorts at a ratio of 7:3. An external validation cohort comprising data from Nanchang Third Hospital with 42 patients collected. Three DL models, namely DL-grayscale, DL-CDFI, and DL-elastography, were proposed to predict SLNM by analyzing grayscale ultrasound, CDFI, and elastography images. Three DL models were compared and evaluated to assess diagnostic performance based on the area under the curve (AUC). The AUCs of the DL-grayscale were 0.855 and 0.788 in the internal and external validation cohorts, respectively. For the DL-CDFI model, the AUCs were 0.761 and 0.728, respectively. The diagnostic performance of DL-elastography was superior to that of the DL-grayscale and DL-CDFI. The AUC of the DL-elastography model was 0.879 in the internal validation cohort, with a classification accuracy of 86.13%, sensitivity of 91.60%, and specificity of 82.79%. The generalization capability of DL-elastography remained high in the external cohort, with an AUC of 0.876, and an accuracy of 85.00%. DL radiomics can be used to classify SLNM in breast cancer using ultrasound images. The proposed DL-elastography model based on elastography images achieved the best diagnostic performance and holds good potential for the management of patients with SLNM.

## 1. Introduction

Breast cancer is a malignant tumor with the highest incidence rate in women worldwide and has overtaken lung cancer since 2020.^[1]^ Early detection of axillary lymph node metastasis (ALNM) is critical for breast cancer management and prognosis. Axillary lymph node dissection can determine the degree of metastasis and improve overall survival. However, the removal of the breast and associated lymph nodes may cause several postoperative complications and a long recovery time.^[2]^ Sentinel lymph node is defined as the lymph node that first receives lymphatic drainage from the primary tumor site. Compared to axillary lymph node dissection, sentinel lymph node biopsy (SLNB) has less trauma and quicker recovery,^[3]^ whereas SLNB has contraindications and is an invasive examination. Therefore, there is an urgent need to develop a noninvasive, accurate, and efficient method to assist the preoperative prediction of sentinel lymph node metastasis (SLNM) in breast cancer.

With the current advancements in ultrasonic radiomics, traditional diagnostic methods for ALNM detection have been improved and supplemented.^[4]^ Several studies have demonstrated a link between ultrasonographic manifestations and ALNM in breast cancer.^[5–7]^ However, most studies rely on traditional radiomics, which requires the manual extraction of image features by radiologists. Effective feature extraction highly depends on the quality of each intermediate result in the image-processing step and requires recursive trail-and-error to obtain satisfactory results.^[8]^ In recent years, deep learning (DL) techniques, represented by convolutional neural networks (CNN), have been widely used in the field of ultrasound image recognition.^[9–11]^ The main advantage of CNN is that they can learn high-dimensional abstract image features automatically from the data and reduce the burden of manual feature selection.^[12]^

The aim of the current study was to develop a DL method that allows fully automated prediction of SLNM in breast cancer. Three models, namely DL-grayscale, DL-color Doppler flow imaging (CDFI), and DL-elastography, were proposed to differentiate SLNM by analyzing grayscale ultrasound, CDFI, and elastography images, respectively. The diagnostic efficiency of DL models was evaluated using internal and external validation cohorts.

## 2. Methods

### 2.1. Study population

**Figure 1.**
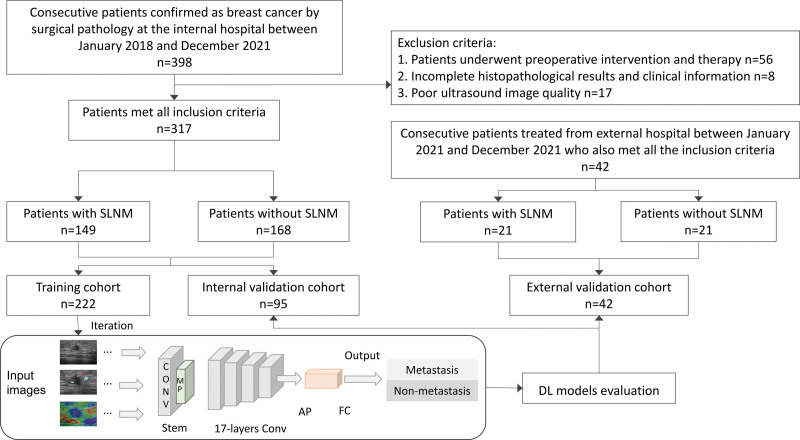
Flowchart of procedures in patients enrollment, and the development and evaluation of the DL models for automated breast cancer SLNM prediction. AP = average pooling layer; Conv = convolution; DL = deep learning; FC = fully-connected layer; MP = max pooling layer; SLNM = sentinel lymph node metastasis.

Between January 2018 and December 2021, medical records of consecutive patients with breast cancer confirmed by surgical pathology were collected (Fig. 1). Inclusion and exclusion criteria were based on the gold standard for pathology. The inclusion criteria were as follows: (1) the patient underwent an ultrasound examination 14 days before surgery; (2) the ultrasound images showed clearly visible lesions that could be used for evaluation; (3) the patient underwent mastectomy and SLNB; (4) postoperative pathological results confirmed breast cancer and whether sentinel lymph node had transferred; and (5) if the patient had a multi-focus breast cancer, only the largest lesion with the highest score of the Breast Imaging Reporting and Data System was included in the analysis.^[13]^ The exclusion criteria were as follows: (1) incomplete histopathological results and clinical information; (2) patients who underwent preoperative intervention and therapy (e.g., radiofrequency ablation, radiotherapy, neoadjuvant chemotherapy); and (3) poor background quality of the ultrasound image such that the tumor could not be recognized.

Subsequent comprehensive pathological reports indicated the presence of breast cancer with SLNM in 149 patients. The construction of DL model needs a balanced database to avoid the overfitting, thus, it has guaranteed system’s pan-ability. Finally, 168 breast cancer patients without SLNM were randomly selected. In the same approach, between January 2021 and December 2021, the external validation cohort was collected using the same methodology from Nanchang Third Hospital, and comprised 42 patients with breast cancer (21 with SLNM and 21 without SLNM).

This study was conducted in accordance with the Declaration of Helsinki (revised in 2013). This study was approved by the Ethics Committee on Biomedical Research of the Second Affiliated Hospital of Nanchang University (No. 20220413112422664). The committee waived the requirement for individual consent for this retrospective analysis because it only involved the use of ultrasound images and did not involve patients’ personal information.

### 2.2. Ultrasound image acquisition and histopathological assessment

Ultrasound examination was performed by 2 radiologists with over 5 years of experience in breast ultrasound. Patients in the Second Affiliated Hospital of Nanchang University were examined using Mindray (Resona 7S; Shenzhen, China) equipped with a 5 to 14 MHz linear array transducer, while patients in Nanchang Third Hospital were examined using a Hitachi (EUB 8500; Tokyo, Japan) equipped with a 5 to 12 MHz linear array transducer. The standard acquisition procedure for grayscale ultrasound images is to obtain longitudinal and transverse sections at the maximum diameter of the lesion, and the lesion is located in the middle of the image. Supplementary images showing any malignant signs of the lesion, such as calcification or structural distortion, were also obtained.^[14]^ During the CDFI examination, a default equipment setting was adopted for all lesions: a scale of 4 cm/s, medium wall filter, and pulse repetition frequency of 700 Hz.^[15]^ One or two CDFI images were obtained in each plane. In addition, breast elastography imaging was immediately performed according to the World Federation for Ultrasound in Medicine & Biology guidelines for performing ultrasound elastography of the breast.^[16]^ Image quality control was performed by a radiologist who worked in consensus according to the exclusion criteria. All images extracted from the database were in the Digital Imaging and Communications in Medicine (DICOM) format. Demographic and clinical data of the patients were recorded.

The detection of sentinel lymph nodes were used interstitial tracer injection (methylene blue). Depending on specific clinical indications, 2 categories of interstitial administration of the lymphatic mapping agent were chosen: deep injection (intratumoral and peritumoral) and superficial injection (intradermal, subdermal, subareolar, and periareolar).^[17]^ Then, all surgical specimens of sentinel lymph nodes were examined by breast pathologists with over 8 years of work experience in hospitals. The status of sentinel lymph nodes was classified as positive (metastasis) or negative (non-metastasis) by pathologists who were blinded to the ultrasonography and clinical results.

### 2.3. Image preprocessing

**Figure 2.**
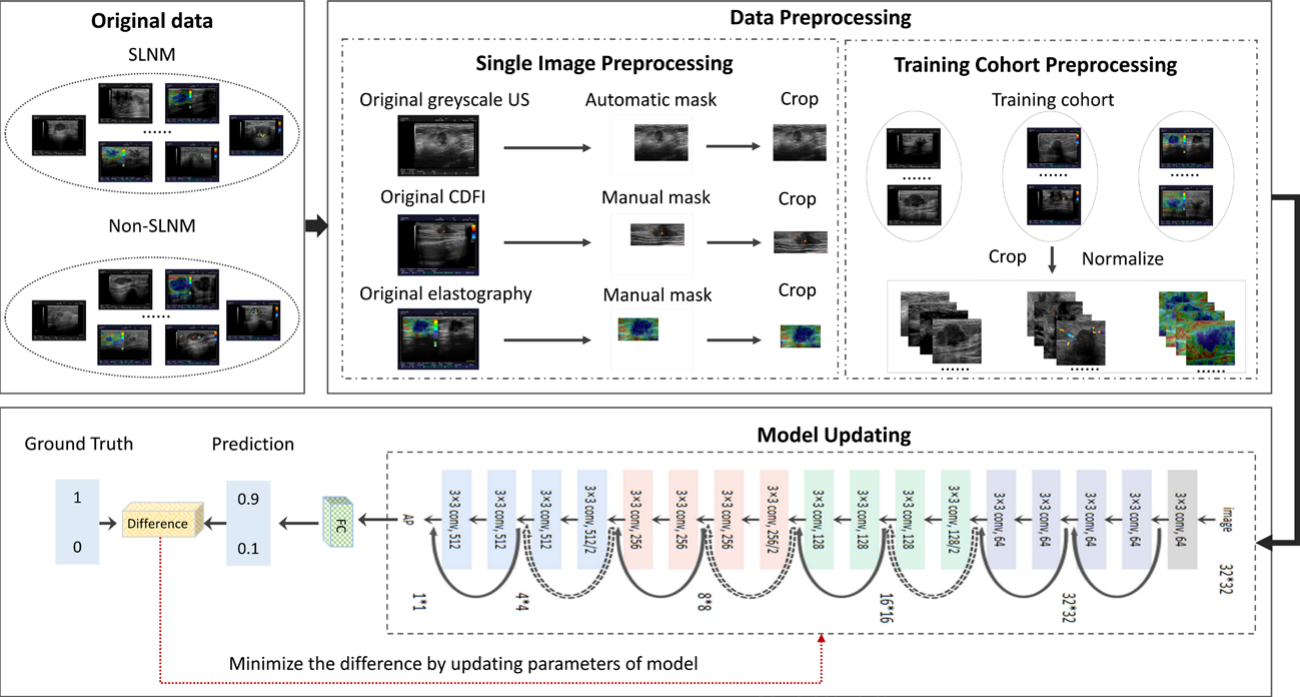
The training process for each individual DL model. AP = average pooling layer; Conv = convolution; FC = fully-connected layer; SLNM = sentinel lymph node metastasis; US = ultrasound.

The software MATLAB (https://ww2.mathworks.cn/products/matlab.html) was selected to convert the DICOM format to JPEG format, and the personal information of images was cropped using homebrew codes. An annotation tool for graphical images, LabelImg (https://github.com/tzutalin/labelImg), was adopted to manually crop the regions of interest of the CDFI and elastography images. Specifically, for each image of the breast lesion, the rectangular box regions of interest was manually delineated and confirmed by one radiologist with more than 5 years of experience in ultrasound examination. The annotation results were saved in JPEG format and parsed using home-brew codes. The grayscale ultrasound images were not further preprocessed. All extracted ultrasound images were normalized to 448 × 448 pixels to standardize the distance scale, which facilitated unification of the input size of the DL models. A detailed flowchart is presented in Figure 2.

The database collected from the Second Affiliated Hospital of Nanchang University was randomly divided into training and internal validation cohorts at a ratio of 7:3. The data collected from Nanchang Third Hospital formed an external validation cohort. There were 1861 grayscale ultrasound images, 1203 CDFI images, and 1000 elastography images obtained from both hospitals.

### 2.4. DL-based radiomics models development

Three DL models (DL-grayscale, DL-CDFI, and DL-elastography models) were proposed to predict SLNM using grayscale ultrasound, CDFI, and elastography images, respectively. To fairly compare the prediction performance, we adopted the same DL structures for DL-grayscale, DL-CDFI, and DL-elastography. In many computer vision tasks, ResNet has been proven to exhibit good stability and excellent performance.^[18]^ To improve the efficiency of the model learning, ResNet-18 was adopted as the basal DL algorithm for model development. Specifically, the construction of ResNet-18 has 3 layers: a convolutional layer, pooling layer, and fully connected layer. The convolutional layer extracts the features of the original breast ultrasound images, and the learned feature map is subsampled by the pooling layer to reduce the feature parameters of the subsequent network layers. Furthermore, the average pooling layer calculated the mean value of the feature map. Finally, the fully connected layer was activated by the softmax function to output the probabilities for each category. In short, the input image features were mapped to the corresponding output label with metastasis and non-metastasis using the model (Figs. 1 and 2).

We trained the DL models with a stochastic gradient descent optimizer running on a computer with one NVIDIA GeForce RIX 2080Ti graphic processing unit and 32 GB of random-access memory. To measure the difference in the classification task between the ground truth and predicted label, the cross-entropy function was selected. In addition, the gradient was back-propagated to update the parameters using the Adam optimizer, with the learning rate decayed from 10^−2^ to 10^−5^ in 100 epochs.^[19]^ The mini-batch size was set to 8.

### 2.5. DL models performance evaluation and statistical analysis

Continuous data with a normal distribution are reported as mean ± SD; data with a non-normal distribution are reported as median (range). Counting data are expressed as frequencies and percentages [n (%)]. Differences in the characteristics of the training, internal, and external validation cohorts were compared using a chi-square test or Student *t* test, where appropriate. All tests were two-sided, and statistical significance was set at *P* < .05.

The probability score of the softmax function and the corresponding tag value were recorded for each sample in the internal and external validation cohorts. The scores were sorted from high to low and a threshold was set.^[20]^ Area under the receiver operating characteristic curve (AUC), sensitivity, specificity, accuracy, positive predictive value, and negative predicted value were used to estimate the diagnostic performance of the DL models.

In our study, model development was achieved using Python (https://www.python.org/downloads/release/python-360/, version 1.6), and PyTorch (https://pytorch.org/docs/stable/index.html, version 1.7.0).^[21]^ Statistical analysis was conducted using the R software (https://www.r-project.org/, version 3.2.3).

## 3. Results

### 3.1. Baseline characteristics

Between January 2018 and December 2021, a total of 398 patients were pathologically diagnosed with breast cancer at the Second Affiliated Hospital of Nanchang University. Among these patients, 81 were excluded for preoperative intervention and therapy as well as unqualified pathological results, clinical information, and ultrasound image quality (Fig. 1). Between January 2021 and December 2021, 42 patients with breast cancer were diagnosed by pathology after surgery at Nanchang Third Hospital. Among the 359 patients, 170 (170/359, 47.4%) had SLNM, and 189 (189/359, 52.6%) did without SLNM.

The characteristics of the training cohort and the internal and external validation cohorts are presented in Table 1. The training, internal and external validation cohorts consisted of 222, 95, and 42 patients, respectively, with ages 48.5 ± 10.4 years, 48.8 ± 10.0 years, and 51.1 ± 10.3 years, respectively. Age (*P* = .916), sex (*P* = .834), clinical mass size (*P* = .298), and pathological type (*P* = .561) did not differ significantly between the training and internal validation cohorts.

**Table 1:** Baseline characteristics of patients.

Characteristics	Training cohort	Internal validation cohort	External validation cohort	*P* value*
No. of patients	222	95	42	–
No. of grayscale US images	1186	504	171	–
No. of CDFI images	812	346	45	–
No. of elastography images	657	281	60	–
*Age, mean ± SD (years*)	48.5 ± 10.4	48.8 ± 10.0	51.1 ± 10.3	.916
Age ≤ 45	71	33	12	
Age > 45	151	62	30	
*Gender*				.834
Male	3	1	0	
Female	219	94	42	
*Clinical mass size*	2.3 ± 0.9	2.2 ± 1.1	2.5 ± 0.8	.298
≤ 2.0 cm	132	64	13	
2.0–5.0 cm	83	28	29	
> 5.0 cm	7	3	0	
*Pathological type*				.561
Invasive ductal carcinoma	135	58	38	
Invasive lobular carcinoma	15	6	1	
Others	72	31	3	

Values are presented as mean ± SD (continuous data).

CDFI = color Doppler flow imaging; US = ultrasound.

* T-test between training and internal validation cohorts.

### 3.2. Evaluation on DL models

**Figure 3.**
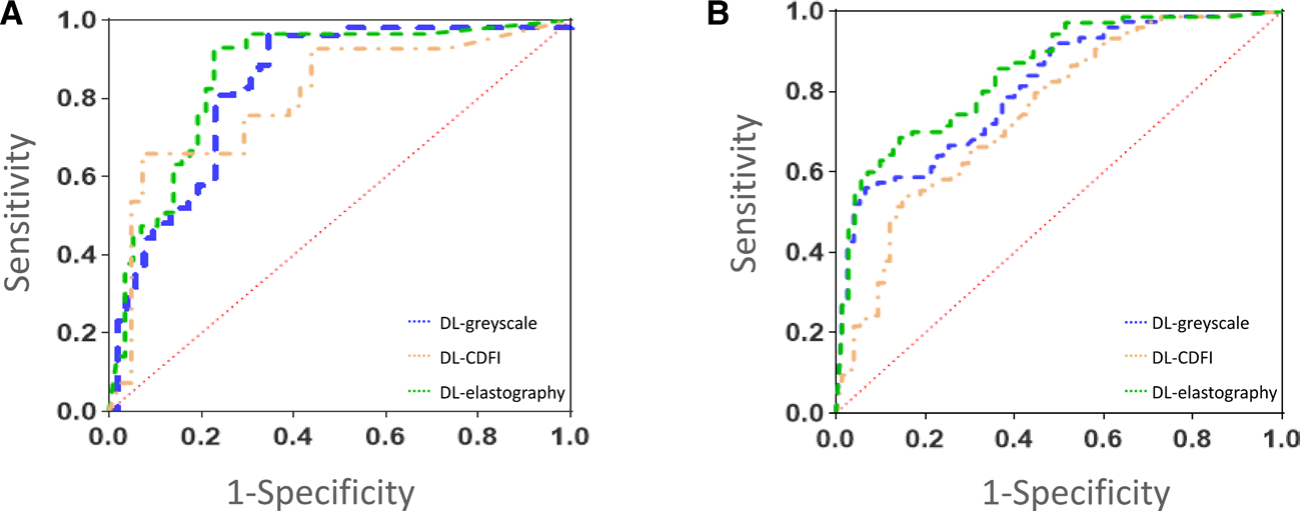
Receiver operating characteristics (ROC) curves of 3 DL models: (A) internal validation cohort of 95 patients. AUC of DL-grayscale, DL-CDFI, and DL-elastography were 0.855, 0.761, and 0.879, respectively; (B) external validation cohort of 42 patients. AUC of DL-grayscale, DL-CDFI, and DL-elastography were 0.788, 0.728, and 0.876, respectively. AUC = areas under the curve; CDFI = color Doppler flow imaging; DL = deep learning.

Table 2 shows the detailed diagnostic performance of the 3 DL models in the internal and external cohorts. The DL-elastography model achieved a high diagnostic performance in both internal and external cohorts. The AUC of DL-elastography were 0.879 [95% confidence interval (CI): 0.839–0.920] and 0.876 (95% CI: 0.813–0.970) in the internal and external cohorts, respectively. In the internal validation cohort, the sensitivity and specificity of DL-elastography were 91.60% and 82.79%, respectively. In the external validation cohort, the sensitivity and specificity of DL-elastography were 88.46% and 82.35%, respectively. For the DL-grayscale model, the AUC were 0.855 (95% CI: 0.819–0.890) and 0.788 (95% CI: 0.716–0.860) in the internal and external validation cohorts, respectively. The sensitivity and specificity in the external validation cohort were 78.49% and 84.61%, respectively. For the DL-CDFI model, the AUC were 0.761 (95% CI: 0.694–0.829) and 0.728 (95% CI: 0.677–0.879) in the internal and external validation cohorts, respectively. In the external validation cohort, the sensitivity and specificity were 72.22% and 70.37%, respectively. Figure 3 shows the receiver operating characteristics curves for the 3 DL models in the internal and external validation cohorts.

**Table 2:** The performance of the DL models.

	AUC	Sensitivity, %	Specificity, %	Accuracy, %	PPV, %	NPV, %
*Internal validation cohort*						
DL-grayscale	0.855 (0.819–0.890)	82.01	84.51	83.13	86.69	79.25
DL-CDFI	0.761 (0.694–0.829)	77.66	71.12	73.31	57.48	86.36
DL-elastography	0.879 (0.839–0.920)	91.60	82.79	86.13	76.43	94.18
*External validation cohort*						
DL-grayscale	0.788 (0.716–0.860)	78.49	84.61	81.29	85.88	76.74
DL-CDFI	0.728 (0.677–0.879)	72.22	70.37	71.11	61.90	79.17
DL-elastography	0.876 (0.813–0.970)	88.46	82.35	85.00	79.31	90.32

AUC = area under the curve, CDFI = color Doppler flow imaging, DL = deep learning, NPV = negative predicted value, PPV = positive predicted value.

**Figure 4.**
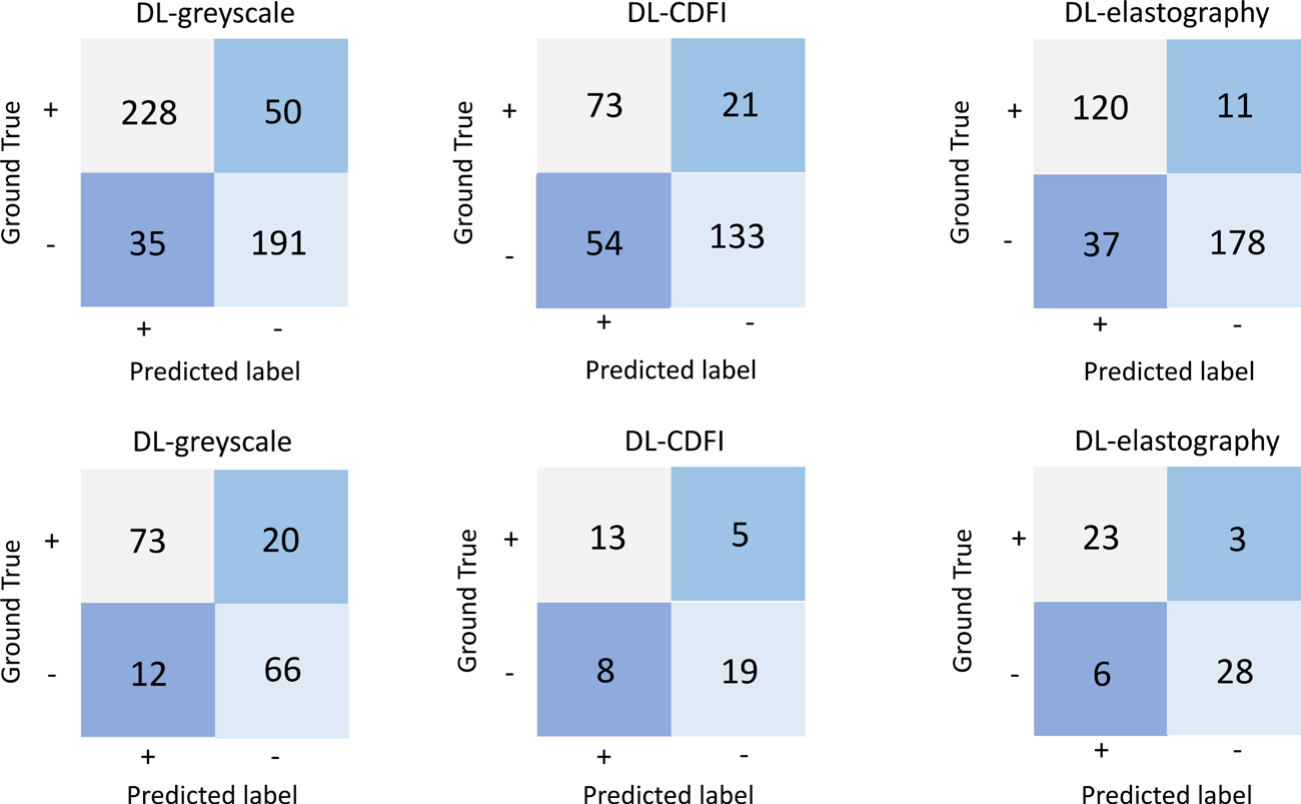
Confusion matrix of internal and external validation cohorts to determine the presence or absence of SLNM using DL models: (A) internal validation cohort; (B) external validation cohort. CDFI = color Doppler flow imaging; DL = deep learning; SLNM = sentinel lymph node metastasis.

**Figure 5.**
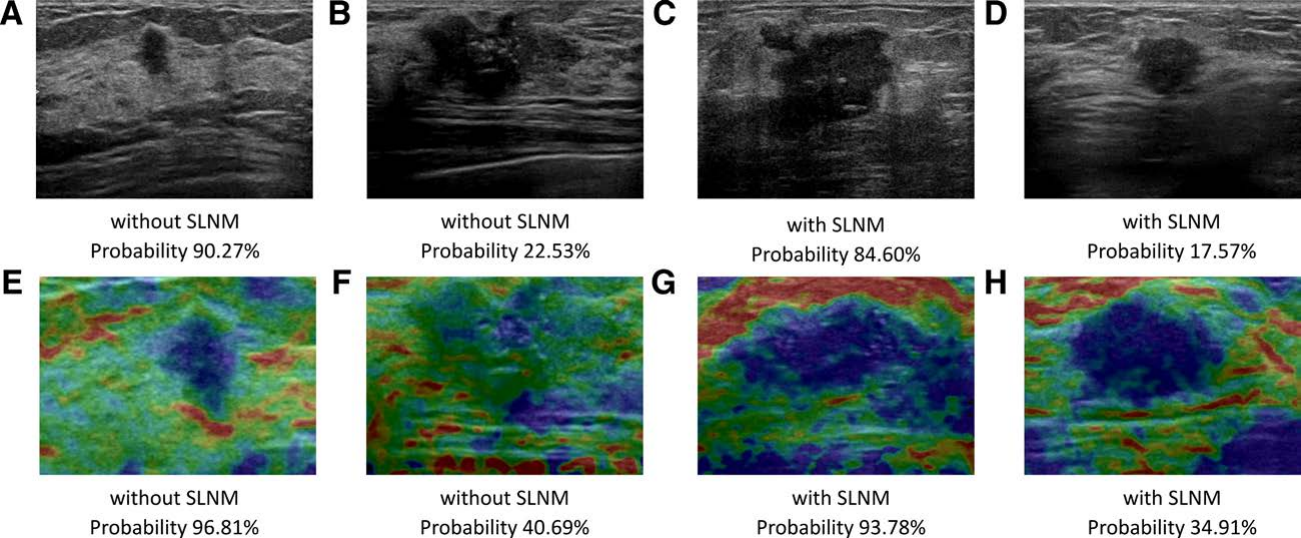
Examples of patients with or without SLNM correctly or incorrectly classified by DL-grayscale and DL-elastography: (A and E) A 49-year-old DCIS patients without SLNM. The probability of DL-grayscale being correctly diagnosed is 90.27%. The probability of DL-elastography being correctly diagnosed is 96.81%; (B and F) a 69-year-old IDC patients without SLNM. The probability of DL-grayscale being incorrectly diagnosed is 22.53%. The probability of DL-elastography being incorrectly diagnosed is 40.69%; (C and G) A 74-year-old IDC patients with SLNM. The probability of DL-grayscale being correctly diagnosed is 84.60%. The probability of DL-elastography being correctly diagnosed is 93.78%; (D and H) A 39-year-old IDC patients with SLNM. The probability of DL-grayscale being incorrectly diagnosed is 17.57%. The probability of DL-elastography being incorrectly diagnosed is 34.91%. DCIS = ductal carcinoma in situ; IDC = invasive ductal carcinoma; SLNM = sentinel lymph node metastasis.

The confusion matrices of the internal and external validation cohorts are shown in Figure 4, including the correct and error classification numbers for each DL model for breast cancer with and without SLNM. The horizontal lines represent the truth of the pathological results and the longitudinal lines denote the prediction of the models. The DL-elastography model showed the best performance in both the internal and external validation cohorts. Of the 48 cases that were incorrectly classified by DL-elastography, 11 with SLNM were incorrectly identified as without SLNM. The positive predictive value and negative predicted value were 76.43% and 94.18%, respectively, in the internal validation cohort. Figure 5 shows examples of DL models that identify the probability of SLNM.

## 4. Discussion

### 4.1. Main findings

In this study, we established different DL models based on grayscale ultrasound, CDFI, and elastography images to identify SLNM in patients with breast cancer. The internal validation cohort showed that the AUC of the 3 DL models ranged from 0.761 to 0.879, whereas the AUC of the external validation cohort ranged from 0.728 to 0.876. The AUC of DL-elastography was higher than that of the DL-gray scale and DL-CDFI in both the internal and external validation cohorts. DL-elastography achieved the best predictive performance with AUC of 0.879 and 0.876 in the internal and external cohorts, respectively. Given the above, we strongly believe that the DL radiomics models proposed in this study based on breast ultrasound images have great potential for clinical application.

### 4.2. The limitations of traditional prediction of SLNM in breast cancer

Breast cancer is a malignant tumor with the highest incidence rate among women worldwide, with the incidence rate increasing by 0.5% annually.^[22]^ Sentinel lymph nodes are regional lymph nodes that drain the lymph directly from the primary tumor. The status of sentinel lymph nodes is one of the most important prognostic indicators of breast cancer and can guide staging and treatment strategies. SLNB is a highly reliable method for screening axillary nodes and identifying metastatic disease in the regional lymph nodes.^[17]^ Furthermore, studies have shown that the risk of axillary recurrence and complications in breast cancer patients with negative/few-positive sentinel lymph nodes omitting ALND is very low.^[23–25]^ Currently, tracers (e.g., methylene blue, indocyanine green, and ^99m^Tc) are used for SLNB. However, owing to the low molecular weights of the tracers, the operation should be completed within a limited time. Besides, SLNB is an invasive surgical procedure. Thus, accurate and noninvasive supplementary diagnostic methods may lead to better treatment outcomes.

Some traditional radiomics studies have demonstrated a correlation between ultrasonographic manifestations and ALNM in breast cancer.^[6,7]^ The traditional radiomics generally needs to manual select different features and assign various values by experienced radiologists. In contrast, the DL algorithm classifies the data by mapping the characteristics from low-dimensional space to high-dimensional space using convolutional layer and softmax function. DL automatically identifies the potential features of images, which can effectively avoid subjectivity and reduce the burden of feature selection.^[26]^ A comparison between CNN (DL algorithm) and random forest (the traditional radiomics) was provided in a study performed on 479 patients with 2395 breast ultrasound images.^[27]^ The results showed that the CNN achieved better performance than the random forests. Sun et al^[28]^ trained and tested a DL model based on grayscale ultrasound images to predict ALNM. The results reported an AUC of 0.72 and an accuracy of 72.6%. The above studies demonstrated that CNN had excellent predictive performance in ALNM. However, these findings were based on grayscale ultrasound images alone and did not involve the prediction of SLNM. Therefore, our study used different types of ultrasound images to train DL models, and collected internal and external validation cohorts to verify the diagnostic performance of DL models in predicting SLNM. The results showed DL models have a great potential for predictive utility.

### 4.3. Strengths of this study

In this study, we innovatively developed 3 DL models to classify SLNM using preoperative grayscale ultrasound, CDFI, and elastography images, respectively. Methodologically, we elaborately adjusted the distributions of the weights based on previous experience,^[21]^ which made the DL models achieve high accuracy while maintaining low complexity. The DL-grayscale model achieved an AUC of 0.855 in the internal cohort, which was higher than that in previous studies.^[27,28]^ Besides, the majority of previous studies only predicted ALNM in breast cancer and had no external cohort to validate the generalization ability. Our study showed an AUC of 0.788 in the external cohort, suggesting that the DL-grayscale has good predictive performance and generalization ability. Tumor growth and metastasis depend largely on angiogenesis.^[29,30]^ The blood supply to the tumor is reflected by CDFI. However, the results of DL-CDFI were unsatisfactory in this study, as they were inferior to the DL-gray scale. We suspected that the characteristics of breast cancer are abundant with the blood supply, and the small discrepancy in the blood flow signal may have influenced the image recognition of the DL model.

Ultrasound elastography is a novel imaging technique for noninvasive assessment of the mechanical properties of tumor tissue elasticity, which has made significant progress in the diagnosis of breast diseases.^[31,32]^ The underlying mechanism of elastography is the characterization of tissue stiffness using ultrasound imaging.^[33]^ In this study, compared with the DL-grayscale model based on grayscale ultrasound images, the DL-elastography model based on elastography images achieved better diagnostic performance. This phenomenon demonstrates that elastography images could provide added value for improving the diagnostic performance of DL models. Biological deformation in tumor tissues may influence radiological characteristics and feature selection in ultrasound images, which can be encoded by the DL algorithm.

### 4.4. Limitations and future study

The present study had several limitations. Firstly, it was a retrospective study based on a limited database. Multicenter prospective and unbalanced database are required to validate the predictive performance and robustness of the radiomics model. Secondly, there was no database to compare the results of DL models and SLNB. Finally, images of the breast lesions were not combined with the axillary lymph nodes. These issues should be addressed in future studies.

## 5. Conclusion

In this study, we designed 3 DL models based on preoperative grayscale ultrasound, CDFI, and elastography images. The proposed DL-elastography model based on elastography images performs the best, which may hold good potential for benefiting the subsequent treatment of breast cancer patients with SLNM.

## Acknowledgments

All authors thank radiologists and patients from the Second Affiliated Hospital of Nanchang University and Nanchang Third Hospital. And we thank Paperpal (https://preflight.paperpal.com) for the English language review of this manuscript.

## Author contributions

**Conceptualization:** Chujun Wang, Yu Zhao, Chun-Quan Zhang.

**Data curation:** Chujun Wang, Yu Zhao, Lingmin Liao.

**Formal analysis:** Min Wan, Long Huang.

**Methodology:** Chun-Quan Zhang.

**Project administration:** Liangyun Guo, Jing Zhang.

**Software:** Min Wan.

**Writing – original draft:** Chujun Wang.

**Writing – review & editing:** Chujun Wang, Yu Zhao, Chun-Quan Zhang.
